# Marine Microorganism-Invertebrate Assemblages: Perspectives to Solve the “Supply Problem” in the Initial Steps of Drug Discovery

**DOI:** 10.3390/md12073929

**Published:** 2014-06-30

**Authors:** Miguel Costa Leal, Christopher Sheridan, Ronald Osinga, Gisela Dionísio, Rui Jorge Miranda Rocha, Bruna Silva, Rui Rosa, Ricardo Calado

**Affiliations:** 1 Departamento de Biologia & CESAM, Universidade de Aveiro, Campus Universitário de Santiago, Aveiro 3810-193, Portugal; E-Mails: gisela.dionisio@gmail.com (G.D.); ruimirandarocha@ua.pt (R.J.M.R.); sbruna@sapo.pt (B.S.); 2 Skidaway Institute of Oceanography, University of Georgia, 10 Ocean Science Circle, Savannah, GA 31411, USA; 3 Biology of Marine Organisms and Biomimetics Laboratory, Research Institute for Biosciences, University of Mons, Pentagone 2B, 6 Avenue du Champ de Mars, Mons 7000, Belgium; E-Mail: Christopher.Sheridan@umons.ac.be; 4 Department of Aquaculture and Fisheries, Wageningen University, P.O. Box 338, 6700 AH Wageningen, The Netherlands; E-Mail: ronald.osinga@wur.nl; 5 Porifarma BV, Poelbos 3, 6718 HT Ede, The Netherlands; 6 Laboratório Marítimo da Guia, Centro de Oceanografia, Faculdade de Ciências da Universidade de Lisboa, Av. Nossa Senhora do Cabo, 939, Cascais 2750-374, Portugal; E-Mail: rarosa@fc.ul.pt

**Keywords:** marine natural products, aquaculture, microbial symbionts, marine invertebrates, pharmaceuticals

## Abstract

The chemical diversity associated with marine natural products (MNP) is unanimously acknowledged as the “blue gold” in the urgent quest for new drugs. Consequently, a significant increase in the discovery of MNP published in the literature has been observed in the past decades, particularly from marine invertebrates. However, it remains unclear whether target metabolites originate from the marine invertebrates themselves or from their microbial symbionts. This issue underlines critical challenges associated with the lack of biomass required to supply the early stages of the drug discovery pipeline. The present review discusses potential solutions for such challenges, with particular emphasis on innovative approaches to culture invertebrate holobionts (microorganism-invertebrate assemblages) through *in toto* aquaculture, together with methods for the discovery and initial production of bioactive compounds from these microbial symbionts.

## 1. Introduction

Marine ecosystems harbor a substantial fraction of Earth’s biodiversity and provide a wide range of goods and services [1]. Among these, marine natural products (MNP) have received special attention in recent years. The main driver for this particular focus on MNP was the urgent need for new chemical diversity to fuel the drug discovery pipeline. This impulse led to a major increase in the discovery of MNP in the past decades [2,3], with over 20,000 new compounds described since the 1950s [4]. A large fraction of these new metabolites were obtained from marine invertebrate species [2], which make up about 50% of all extant non-microbial marine biodiversity in the oceans [5]. Although success stories of marine derived-drugs are already a reality [6], the true potential of MNP from invertebrates as future drug candidates is yet to be unraveled [7,8]. However, one critical issue usually associated with the initial steps of marine drug discovery from invertebrates is the lack of a constant and reliable supply of animal biomass [9]. Relevant to this supply problem is the realization that symbiotic microorganisms, including protists, may, in fact, synthesize a large number of metabolites once considered to be produced by marine invertebrates [9,10,11,12].

The present review addresses the potential of MNP derived from microorganisms-invertebrate assemblages and the production of the target metabolites for the initial steps of drug discovery. This work focuses on *in toto* aquaculture of invertebrate holobionts, *i.e.*, the invertebrate host and the associated community of microorganisms, as well as on culture-dependent and independent strategies for the isolation of bioactive compounds from microbial symbionts for drug discovery. Although aquaculture for drug discovery is still an emerging field, particular emphasis is also given to the manipulation of culture conditions that may contribute toward maximizing the production of microbial symbionts and their secondary metabolites.

## 2. Marine Microorganism-Invertebrate Assemblages

Marine invertebrates are a source of high microbial abundance and diversity (e.g., [13,14,15]). For instance, the number of bacteria in sponges may exceed bacterial concentrations in seawater by two to four orders of magnitude [16]. Cnidarians, especially corals, also harbor an impressive number of microbial organisms; for example, the coral mucus may reach microbial concentrations 100- to 1000-fold higher than those observed in seawater [17]. Invertebrate microbial symbionts are also highly diverse [18]. The abundant and unique symbiotic microbial diversity hosted by marine invertebrates [18,19] plays a very important role in their biology and ecology, particularly in its nutrition [20,21,22], disease-resistance [13,17] and response to environmental perturbations [23,24]. Symbiotic microorganisms are also known to be active players in the chemical mediation of interactions among marine organisms [25].

Marine microbial symbionts have been recognized for their active role in chemical defenses of marine invertebrates against both predators and competitors [25,26,27]. The symbionts produce chemically diverse and biologically active secondary metabolites, such as anti-inflammatory, antibiotic, antitumor, anticancer, antibacterial and antifungal compounds, whose properties are particularly interesting for drug discovery [6,11,28]. The microbiome of marine invertebrates may represent a remarkable proportion of the holobiont biomass. In sponges, for instance, the microbial community may contribute up to 60% of the holobiont biomass [29]. Contrastingly, in other invertebrates, such as scleractinian corals, symbiotic microorganisms are highly abundant in the soft tissue of the host, but represent a minor fraction of the total biomass, due to the weight that their hard calcium carbonate skeleton represents [30]. However, regardless of microbial abundance, the compounds produced by symbiotic microorganisms of marine invertebrates with potential for drug discovery are usually secondary metabolites. As such, these compounds are naturally produced in low quantities. This imposes critical challenges to the drug discovery pipeline, particularly during the early phases of discovery and the selection of which leads should advance to the next step [31,32,33].

## 3. The Supply Problem in Marine Drug Discovery

While in the past, the search for new MNP heavily relied on the harvesting of wild specimens and large quantities of animal biomass were needed to screen for new chemical diversity, nowadays, small amounts of tissue from a single individual can be enough for an initial screening [32,34]. Nonetheless, this dependence on natural samples may still entail replicability issues [32]. Wild marine organisms collected for bioprospecting are exposed to environmental variability, as well as changes at the community level, which may significantly affect their chemical ecology [35]. Individuals of the same species sampled in different areas, or time frames, may not display the same chemical composition [36] and, therefore, fail to guarantee the supply of a target metabolite (a pitfall commonly termed “loss of the source”). This may also be a potential caveat for the initial detection of bioactive metabolites, as environmental and individual variability in the chemical composition of target organisms may bias bioprospecting [32]. Also associated with replicability issues is the potential loss of the source through extinction of target species. This issue is particularly relevant in the oceans of today and tomorrow, as vulnerability to extinction in marine ecosystems is predicted to be higher on tropical coral reefs [37,38], which have been bioprospecting hotspots since the 1990s [2].

The relatively low natural abundance of bioactive metabolites that is often recorded in marine invertebrates [39] is not a constraint for the initial steps of the drug discovery pipeline, as only small amounts of biomass are currently required. However, while the amount of pure compound required for screening an isolate is usually lower than 1 mg, an increase in several orders of magnitude of target compound quantity (e.g., a few g) is needed to progress satisfactorily towards preclinical trials [34]. Once the compound proceeds through clinical trials and is commercialized as a pharmaceutical, kilograms of the pure compound are required to supply drug production, which may correspond to an annual capture or production of several tones of the invertebrate holobiont [33,34]. Overall, the challenges associated with the harvest/culture of target organisms to yield either milligrams, grams or kilograms of a given pure compound inevitably triggers supply issues that only escalate as the compound progresses into later development stages of the drug discovery pipeline [40]. Therefore, the production of these compounds at a scale large, enough to fulfill the needs of drug discovery and potential commercial applications, has been a major issue [6,40] that has prompted innovative solutions. To overcome the “supply problem”, current R&D strategies of the pharmaceutical industry are largely associated with the development of synthetic or hemisynthetic analogues and the use of heterologous gene expression techniques [41], as well as with the design of molecules displaying a lower complexity and a similar bioactive function that can be synthesized using standardized techniques [33,42]. However, there are several constraints associated with these approaches. The remarkable complexity of certain natural molecules (most are chiral and display intricate structures) makes it very difficult, and often impossible, to replicate the natural molecule in the laboratory [40,43]. Furthermore, the large number of steps often required to produce such synthetic analogues, together with the notable number of misassigned products, commonly represent an extreme financial burden that most drug discovery companies are unable to support [44]. Hypothetically, even if chemical synthesis was a technically feasible option, the production of the target metabolite at the kilogram scale would probably not be affordable for commercial applications [45]. While heterologous gene expression is a very useful and promising technique for drug discovery from symbiotic microorganisms [31], it is important to note that target genes from source organisms might not be expressed in all hosts, and therefore, developing such an assay for new species requires considerable efforts. Moreover, the target gene from the symbiotic microorganism may need a cue to trigger its expression, such as the influence of other community members, or share a metabolic pathway with its invertebrate host [46].

It is therefore undeniable that the “supply problem” is at the center of the main constraints impairing drug discovery from the marine environment and is usually strongest at the early stages of drug development [6,32,40]. This issue becomes even more relevant when target compounds are produced by symbiotic microbes due to the low levels at which these metabolites are produced, along with the smaller proportion that microbial biomass commonly represents when compared to the majority of their invertebrate hosts [10,11,31]. Nevertheless, the interest in the remarkable properties of MNP remains appealing enough to inspire innovative solutions to the supply problem [6]. The *in toto* aquaculture of the holobiont [47] and the culture of symbiotic microorganisms present in the microbiome of invertebrates [48] are certainly promising approaches to find potential solutions for such bottlenecks.

## 4. Aquaculture of Marine Invertebrates

**Figure 1:**
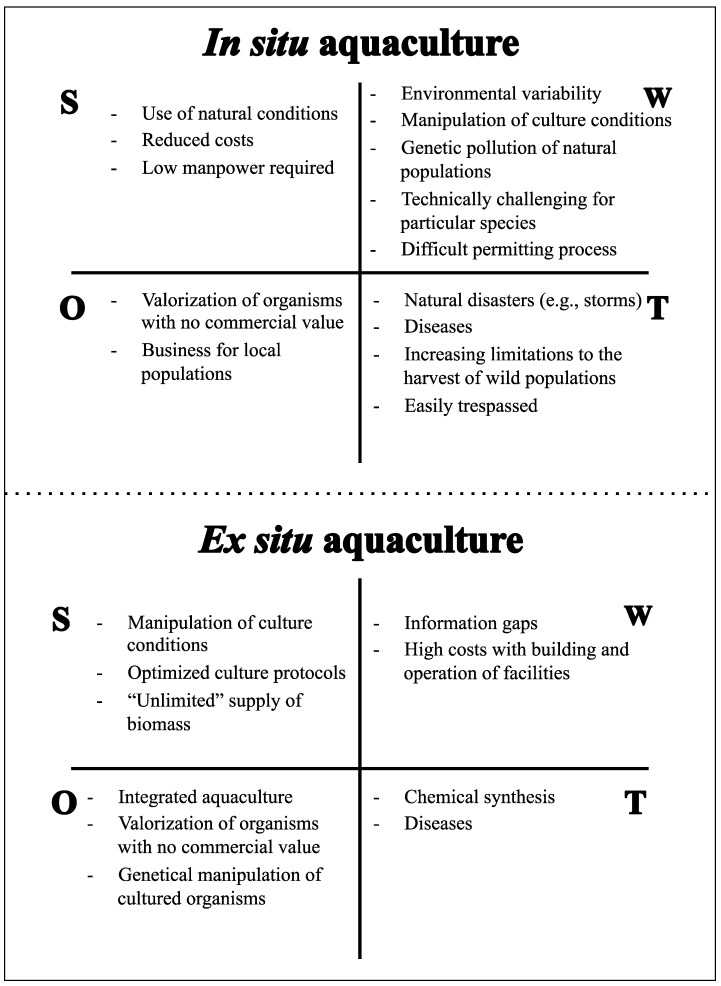
SWOT analysis (strengths, weaknesses, opportunities and threats) of the *in situ* and *ex situ* aquaculture of marine invertebrates for marine drug discovery and development.

Current aquaculture practices can be broadly classified as *in situ* or *ex situ*. This terminology is mostly associated with the production site. *In situ* aquaculture, also known as mariculture, is the culture of organisms in the marine environment using natural conditions. *Ex situ* aquaculture is the process of producing organisms in a controlled environment. Both have advantages and disadvantages (Figure 1). *In situ* aquaculture entirely relies on natural conditions (water physical and chemical parameters, water flow, current and hydrodynamics, light and nutrients) required for the propagation and growth of the target species and requires no adaption to an artificial propagation system. However, cultured species can be potentially exposed to several deleterious factors present in the natural environment, such as sedimentation, unfavorable meteorological conditions, predators, parasites, competitors and other natural hazards, which can reduce survival and growth [49]. Human resources play a major role in the assemblage and maintenance of infrastructures, with the most manpower being necessary to kick-off the culture process and to harvest cultured specimens at the end of production cycles. The ability to manipulate culture conditions is, however, fairly limited, as the largest allowed flexibility is the selection of the location where production structures are implemented. This decision is extremely important, as different areas may display contrasting environmental conditions and thus affect the success of *in situ* aquaculture [50]. In contrast, *ex situ* aquaculture requires a more skilled work force and has higher costs associated with the building and operation of culture facilities. Nonetheless, the ability to manipulate biotic and abiotic factors to maximize animal biomass and metabolite production is incomparably higher than that for *in situ* aquaculture. *Ex situ* aquaculture allows the use of optimized husbandry methodologies specifically designed for the target species being produced. Additionally, and unlike *in situ* aquaculture, *ex situ* production prevents the risks of genetic pollution of natural populations associated with the mass culture of single genotypes in the wild [51]. Nonetheless, *ex situ* aquaculture techniques may also have impacts on the natural environment, which should be prevented or minimized through proper regulation of effluents that may be loaded with nutrients and chemicals used for therapeutic purposes and water-quality management [52]. However, it should be noted that *ex situ* aquaculture of invertebrates for non-food purposes often uses recirculation techniques specifically designed to minimize the discharge of waste water.

Despite the technological simplicity of production *in situ* and the correspondingly expected low production costs, this approach has proven to be more technically challenging and expensive than previously assumed for supporting drug discovery [53]. While incurring higher production costs and requiring skilled workers for the implementation and maintenance of production systems, *ex situ* aquaculture can be implemented in privileged locations, such as in the vicinity of pharmaceutical laboratories; this physical location can ensure a more rigorous processing after the harvesting of produced specimens and, thus, avoid costs associated with paperwork, handling, packaging, shipping and spoilage during the transport of produced biomass.

The drawbacks associated with *in situ* aquaculture (Figure 1) may be overcome through *ex situ* aquaculture in controlled environments, which may eliminate problems commonly faced by researchers, such as the loss of the source and reproducibility. Additionally, in *ex situ* cultures, environmental conditions can be manipulated, optimized and stabilized to: (i) accelerate the growth of cultured species; (ii) ensure the presence of symbiotic taxa in the invertebrate host that are known to be important producers of the target compound (e.g., *Aspergillus* spp. in sponges) [54,55,56,57]; (iii) increase or decrease the number of symbionts according to their relevance in metabolite production; and (iv) adjust the biotic and/or abiotic settings that ensure the maximum yield of the target bioactive compounds [58]. *Ex situ* aquaculture further allows better control of the genetic selection of the target species, which ultimately leads to the optimization of culture conditions for particular genotypes that may yield higher metabolite production. Specific genotypes may also hold contrasting microbial communities with different metabolite production.

The use of aquaculture for drug discovery is still in its infancy, and *in toto* production of marine organisms is a process that deserves greater attention from researchers aiming to produce the biomass of secondary metabolites [47]. The use of controlled environments *ex situ* can minimize environmental variability that affects the chemical ecology of the holobiont and, consequently, contributes to achieving a higher degree of replicability. This feature is of paramount importance to maintain a stable community of bioactive metabolite-producing symbiotic microorganisms in the invertebrate host. Furthermore, as previously mentioned, culture protocols may also be optimized to continuously provide animal/microbial biomass and maximize target metabolite production [58].

The following sections focus on aquaculture practices of different invertebrate groups and the optimization of culture protocols that can maximize symbiont biomass and metabolite production, with emphasis on successful case studies. The invertebrate groups emphasized here are sponges, cnidarians, mollusks, as well as a few other taxa relevant for drug discovery (e.g., bryozoans, tunicates and worms). This selection is based on the importance of these groups in MNP discovery [2], as well as on ongoing efforts to address the aquaculture of these marine invertebrate groups [59,60,61,62].

### 4.1. Sponges

For many years, sponges (Porifera) have been regarded as the primary target for MNP discovery [2]. Sponges illustrate the supply issue at its best, as their MNP are biochemically complex (*i.e.*, difficult targets for chemical synthesis) and are often present only in minute quantities in animal tissues. Sponges are also known to host a large and diverse community of microorganisms [63], which can compose a notable fraction of the sponge tissue volume [16]. Such symbiotic microorganisms may display interesting bioactivities as new drug leads. For instance, marine-derived fungi, such as *Aspergillus ustus* and *Petriella* sp., were isolated from the sponge, *Suberites domuncula*, and yielded cytotoxic sesquiterpenoids that have anticancer applications [64,65]. Moreover, bacteria within the genera, *Aquamarina*, *Pseudovibrio* and *Streptomyces*, displaying anti-fungal and anti-bacterial activity, were isolated from the sponges, *Amphilectus fucorum* and *Haliclona simulans* [66].

Many sponge species with the potential for drug discovery are not available in large quantities in nature, and sponges are notoriously difficult to culture [67]. A well-known example is the anticancer compound, halichondrin B, isolated from *Halichondria okadai*, which is now a drug (Halaven^®^) and was based on a synthesis of the active end following an initial biomass supply from New Zealand, with the original compound being present in quantities lower than 1 mg∙kg^−1^ of wet sponge biomass [41]. This example clearly shows that reliable methods for the production of sponge materials are desired to fully explore the possibilities that sponge MNP have to offer.

**Figure 2:**
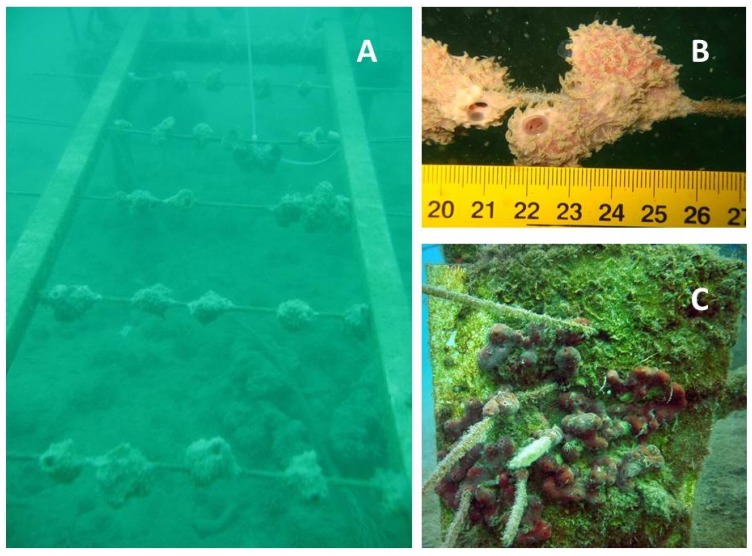
Examples of Mediterranean sponges in sea-based aquacultures. (**A**) Culture frame with spike-cultures of *Dysidea avara*, as described in Osinga *et al.* [78]. In addition, many sponges primarily feed on dissolved organic carbon (DOC) rather than being particle feeders [72,73,74,75], which has altered the view on designing feeding regimes for sponges in aquaria; (**B**) Detail of *D. avara* growing on spikes; (**C**) Culture of *Chondrilla nucula* on vertical plates. Images by M. Gokalp and R. Osinga.

Scientists have substantially increased their efforts to develop culture techniques for marine sponges, which include *in situ* aquaculture (reviewed by [68]) and *ex situ* culture approaches, such as aquarium culture, primmorphs and cell cultures (reviewed by [67,69]). While sea-based culture techniques (Figure 2) have proven to be feasible for products, such as halichondrin [70], avarol [61] and discodermolide [71], *ex situ* approaches have not yet been very successful, mostly due to the lack of scientific knowledge on sponge biology. Sponges have a high potential for growth, but release most of their productivity through the shedding of cellular materials into the environment [72,73,74,75,76].

Apart from general methodological issues, such as choices of culture materials, site selection, cutting methods and explant sizes, recent studies have also included more advanced aspects, such as heredity [68]. A clear genotype-associated difference in performance (*i.e.*, growth and metabolite production) was observed for *Discodermia dissoluta* [71]. Such genotypic differences suggest that broodstock optimization through genotypic selection can help increase sponge mariculture productivity. Notwithstanding this, negative effects of repetitive cloning have been reported [49], which may counteract the benefits of genotypic selection. Future studies are therefore required to further elaborate on this aspect.

Most sponges in mariculture retain their ability to produce the compound of interest [49,50,71,77,78,79]. Cultured explants of *Discodermia dissoluta* even contained higher amounts of discodermolide than their wild conspecifics [71]. Attempts to enhance secondary metabolism in mariculture included stress treatments, which were successfully applied to increase the production of avarol [80] and latrunculin B [81]. Other factors reported to influence metabolite concentration include location, depth and seasonality [82,83].

### 4.2. Cnidarians

Cnidarians have become the second most bioprospected group of marine invertebrates [2]. Besides the bioactive metabolites associated with cnidarians [7], there are also important metabolites produced by symbiotic microorganisms associated with corals, such as the fungi, *Nodulisporium* sp. and *Zygosporium* sp., associated with unidentified corals (reviewed by [84]), *Vibrio* species isolated from the soft coral, *Sinularia polydactyla* [85], *Chondrostereum* sp. isolated from the soft coral, *Sarcophyton tortuosum*, and *Aspergillus versicolor* from the soft coral, *Cladiella* sp. (reviewed by [86]), among others. Despite the remarkable chemical diversity and potential for drug discovery displayed by this group of invertebrates, to the best of our knowledge, no metabolite from cnidarians has yet reached the pharmaceutical clinical pipeline, *i.e.*, entered clinical trials or obtained approval for commercialization.

Most efforts on cnidarian aquaculture have been focused on corals (e.g., [87,88]) and sea anemones (e.g., [89]). Studies on *in situ* production methods have primarily focused on asexual propagation through fragmentation [90] and the selection of the area for the grow-out of coral fragments [91,92,93]. The *ex situ* culture of cnidarians is usually performed in tanks with controlled environmental settings: physico-chemical parameters, water flow, light spectra and intensity, as well as feeding. All of these parameters are critical to maximize survival, nutritional/physiological condition and growth [62,89,94,95,96,97,98,99]. Micropropagation techniques have also been developed and create progeny from tissue explants derived from a single polyp of colonial corals [100]. Sexual reproduction of cnidarians is becoming increasingly popular as a method for the production of a large number of organisms, particularly corals [101]. However, research efforts on coral sexual reproduction have been targeting reef restoration and management of captive populations [102,103].

Environmental factors, such as light [58], are important drivers of metabolite production in cnidarians. To our knowledge, only a single study assessed the effect of light intensity on metabolite production (flexibilide) by the soft coral, *Sinularia flexibilis* [58]. The use of herbivorous fishes in the production tanks may also contribute to the production of secondary metabolites that cnidarians use as chemical defense mechanisms [104]. Furthermore, the polyculture of different cnidarian species within the same tank may also induce metabolite production. For instance, chemical extracts from the stony coral *Tubastraea faulkneri* kill larvae of other coral species [105]. Thus, the combination of different coral species, either stony or soft corals, is likely to trigger the production of bioactive metabolites, but may also induce coral mortality [106].

Specimen selection is also an important factor to consider, as the production of secondary metabolites may show sex-specific and inter-clonal variability [106]. Genotypic differences may also affect the composition of the associated microbiota. Symbiotic microorganisms are mainly present in the soft tissue and/or mucus produced by cnidarians, and therefore the increase of the proportion of soft tissue and mucus to the total holobiont biomass may represent a significant increase in the yield of the target metabolite for drug discovery. Although the drivers of coral tissue growth are still poorly investigated, it is known that heterotrophic feeding maximizes coral tissue growth [96,107] and that mucus production is associated with protection from UV, desiccation and increased sediment loading [108].

While cnidarian aquaculture is already an established technology that allows the production of monoclonal organisms [89,99], it has hardly been applied for drug discovery research. Nevertheless, production costs are likely to be affordable to pharmaceutical companies [99] and may even decrease with the use of new technologies, such as LED illumination, that have a lower energetic consumption [96]. *Ex situ* culture systems may also be used to either keep a live library of cnidarians and its associated microbial community or to produce the target organism synthetizing the desired metabolite.

### 4.3. Mollusks

Approximately 1100 distinct compounds have been isolated from less than 300 species of mollusks, and most of them have been isolated from gastropods [4]. The origin of their NP has been attributed to their ability to bio-accumulate or biotransform molecules acquired through feeding [109], *de novo* synthesis [110] and bacterial symbionts [33]. While some mollusk species are protected by shells and an arsenal of peptide toxins, they also host symbiotic bacteria that contribute to their chemical defenses, a feature that holds great interest for marine drug discovery [111]. Shell-less mollusks, such as the sacoglossans, *Elysia* spp., also associate with a number of bacteria that provide chemical defense, which are usually found in their gut and mucus [112,113]. Such mollusks have also been an important source of a very potent class of cytotoxic peptides—the kahalalides [114,115]. For example, cyanobacteria of genera *Symploca* and *Lyngbya* associated with the aplysiid *Dolabella auriculata* produce Dolastatin 10, an antimicrobial peptide [116,117] that is currently in phase II clinical trials as an anticancer agent [118]. Bacteria, such as *Mycoplasma* spp. and *Vibrio* spp., that are known to associate with the sacoglossan, *Elysia rufescens*, produce elisidepsin (Irvalec^©^), another cyclic peptide belonging to the kahalalide family that is currently under phase II development [43]. Actinomycetes represent another example of biochemically interesting symbionts from mollusks. Actinomycetes living in close association with cone snails (*Conus* spp.) contribute to the production of conotoxins, which are peptide-based neurological toxins with analgesic properties [119,120,121,122]. Ziconotide (Prialt^®^), the synthetic equivalent of a naturally occurring 25-amino acid peptide (ω-conotoxin MVIIA) derived from the venom of a predatory cone snail, was the first marine drug to be approved for clinical use [122] to treat chronic pain. Kurasoins, soraphinol C and other molecules with important neuroactive biological properties have also been isolated from three different species of cone snail (*Conus rolani*, *C. pulicarius* and *C. tribblei*) [123,124].

Although the supply problems of these success stories have been mostly overcome through the chemical synthesis of target molecules, many more metabolites from mollusks and their symbiotic microbiota could be on a more advanced stage of the drug discovery pipeline if the supply issue were solved, particularly at the initial steps of drug discovery.

*In situ* aquaculture of mollusks has been widely applied to bivalves for food production [125]. Although there were a few NP discovered in marine bivalves [2], there are still no endeavors developing the *in situ* aquaculture of bivalves for drug discovery. As opposed to bivalves and in contrast to other invertebrate groups, such as sponges and cnidarians, most efforts toward the aquaculture of gastropods have been undertaken *ex situ*. While new advances have been achieved in the last decades in the larviculture and grow-out of marine snails for human consumption and restocking purposes (e.g., [126,127]), large-scale production for biotechnological applications is yet to be addressed. Sea slug production is still restricted to *Aplysia californica*, which is a widely used model organism in neurosciences research [128]. Nonetheless, recent data using small-scale recirculating culture systems opens good perspectives for the production of several other sea slug species and their associated microbiota [60]. The most important environmental parameters to successfully culture sea slugs are associated with water physico-chemical parameters (e.g., temperature, pH, light cycle) and nutrition [129,130,131].

### 4.4. Other Invertebrates

Bioprospecting efforts targeting marine invertebrates have clearly been biased towards sponges and cnidarians [2]. Nonetheless, other groups of marine invertebrates, such as bryozoans, tunicates and nemertine worms, have also yielded promising compounds to fuel drug discovery programs. For example, one pharmaceutical already being commercialized (Yondelis^®^) was inspired from trabectedin, a secondary metabolite from the tunicate, *Ecteinascidia turbinata*, whereas the NP plitidepsin (Aplidin^®^), described as being from the ascidian (tunicate) *Aplidium albicans*, is now under clinical evaluation [132]. Moreover, 3-(2,4-Dimethoxybenzylidene)-Anabaseine (DMXBA; also called GTS-21) is an anabaseine derivative from the hoplonemertine worm, *Amphiporus lactifloreus*, that is in clinical development to treat Alzheimer’s and schizophrenia [133]. It is also worth highlighting that both tunicates and bryozoans have already been targeted by aquaculture efforts aiming to supply drug discovery [134].

The bryozoan *Bugula neritina* has been the focus of pharmaceutical companies due to its NP bryostatin 1, an antineoplastic agent [135]. The first aquaculture efforts for this species employing an *ex situ* approach failed, due to the lack of adequate food provisioning and a constant larval supply [53]. The latter prompted the collection of mature wild organisms to spawn in the laboratory [136] and the improvement of culture protocols [137]. Asexual reproduction of bryozoans through fragmentation, as previously described for corals, has also been described [138]. Issues associated with constant and reliable *ex situ* production promoted the development of *in situ* culture approaches [53,136], which were successfully employed to produce this bryozoan and yield bryostatin 1 within the normal range for ocean populations (average 7.5 μg/g dry weight) [136]. It is further important to note the important role played by symbionts in the biosynthesis of other bioactive metabolites in bryozoans. *B. neritina* is one of the best-documented examples of bioactive metabolite symbiosis, and other bryostatins discovered in this bryozoan species are, in fact, biosynthesized by its bacterial symbionts (reviewed by [139]). 

The tunicate, *E. turbinata*, also represents an interesting case study for aquaculture. Its most potent NP, trabectedin, is present at very low yields (0.0001%), resulting in nearly 1 ton of animal biomass being required to isolate 1 gram of this compound [39,140]. Initial aquaculture efforts were performed both *in situ* (using PVC structures with ropes where lab-settled colonies were attached) and *ex situ* (in tanks) [136]. During the development of Yondelis^®^, the developing enterprise addressed the supply problem with both *in situ* and *ex situ* aquaculture. This approach allowed them to pursue the clinical development of the drug, but aquaculture was revealed to be unfeasible for commercialization due to high production costs and low metabolite yield [140].

These two case studies highlight the importance that aquaculture may play in the production of metabolite biomass from bryozoans and tunicates for drug discovery, at least in the early steps of the discovery pipeline. Although these NP (bryostatin 1 and trabectedin) are produced by the invertebrate host, aquaculture practices may also be used to yield metabolites produced by symbiotic organisms [31]. It is also important to note that the aquaculture of *B. neritina* and *E. turbinata* has mostly been focused on the maximization of biomass production, without any effort to manipulate culture conditions that may increase the production of target metabolites. Stress experiments may contribute toward understanding the drivers of secondary metabolite production and optimizing aquaculture protocols to magnify the yield of target molecules [53,58].

## 5. Discovery of Bioactive Compounds from Marine Invertebrate-Associated Microorganisms and Their Production

The isolation of a pure culture of target microorganisms can be extremely challenging and is often a necessary step in the drug discovery pipeline [141]. Traditionally, microorganisms can be cultivated through serial plating on selective growth media. However, when using these techniques, only 0.001%–1% of the total microbial diversity can be successfully cultivated [142]. These numbers are certainly far from providing an acceptable overview of the microbial landscape of the source environment [143]. When comparing marine and terrestrial environments, the first is highly unstable, heterogeneous and oligotrophic. These conditions make the marine environment particularly difficult to mimic for the cultivation of marine microbes. Some of the constraints that researchers have to face when addressing this issue include: (i) the inability to reproduce highly complex nutritional and environmental conditions, as well as complex networks of cell-cell interactions (e.g., metabolites exchange and signaling); (ii) the overgrowth of slow- by fast-growing microorganisms; (iii) viral infections; and (iv) insufficient time allowed for growth [47,142,143].

Recent breakthroughs have allowed the cultivation of microorganisms previously labeled as unculturable [144]. Initial attempts consisted in optimizing traditional cultivation methods through modifications of culture media (e.g., carbon sources, electron acceptors, nutrient concentration) and growth conditions (e.g., inoculation size, temperature, pH, incubation time) [144,145]. For example, the use of low substrate/nutrient concentrations combined with extended growth time has allowed the cultivation of formerly unculturable bacteria from the taxa Verrucobacteria, Actinobacteria, Acidobacteria and Proteobacteria [146]. Such optimizations of culture conditions and media have allowed significant increases in the propagation of microbial colonies of the culturable fraction of bacteria [147]. However, successful microbial development generally requires interactions between microbes and their environment (*i.e.*, their invertebrate host for microorganism-invertebrate assemblages), as well as with other members of the community. Such interactions include the exchange of metabolites and signals (chemical cues), which is incompatible with traditional pure culture isolation methods [148]. New techniques developed to overcome this issue consist of simulating the conditions of the source environment, either through co-culture (simulating microbial interactions) or *in situ* cultivation (simulating both environmental conditions and microbial interactions). 

The co-culture approach, which allows the development of colonies of microorganisms generally able to grow only in combination with other microbes, has led to the successful isolation and/or enrichment of several previously unculturable microorganisms (e.g., [149,150]). However, while these techniques allow interactions between different members of the cultured communities, they do not simulate/provide other variables of the source environment. A variety of *in situ* cultivation techniques based on diffusion chambers and encapsulation have been proposed in order to fill this gap (see [143,144,147] for a review). Systems based on diffusion chambers have been developed in diverse varieties (e.g., [151,152,153]) and allow communication between the cultured organisms and their environment. Although initial model systems [153] were labor-intensive and low-throughput, the methods developed subsequently were much more efficient and allowed the simultaneous *in situ* cultivation of micro-colonies in up to 96 [152] or 384 [151] diffusion chambers. This approach resulted in the culture of a significantly higher microbial diversity than that commonly achieved using “traditional” techniques [154].

An alternative technique, initially developed by Zengler *et al.* [141], involves the high-throughput cultivation of microorganisms individually encapsulated in agar gel microdroplets (GMDs). While this method was particularly appropriate for the cultivation of slow-growing organisms, as they were protected from overgrowth by fast-growing organisms, the lack of protection from the environment prevented its use for incubation *in situ* [48]. Ben-Dov *et al.* [48] proposed an improvement on GMDs by encapsulating similar agar spheres in a polysulfonic polymeric membrane permeable to nutrients and cues from the environment. This “double encapsulation” technique allows for cell-to-cell interactions and exchanges between the microorganisms and their environment and resulted in the successful *in situ* isolation of previously unidentified microorganisms, including bacteria, fungi and stramenopiles [48].

Whereas the *in situ* cultivation techniques described above are extremely promising, much of the microbial diversity remains to be explored, leaving untapped a huge array of secondary metabolites potentially useful for the development of new drugs. Culture-independent methods may help fill this gap in a variety of ways. Metagenomics can be used to express genes from environmental DNA samples in appropriate vectors, which can then be screened for new compounds of interest through sequence mining or functional expression [155]. Such heterologous gene expression has been successfully used to produce bioactive compounds of pharmaceutical interest from marine organism-associated microbial symbionts, thereby proving the potential of this technique for culture-independent production of bioactive compounds. For example, patellamides A and C, promising compounds for their moderate cytotoxicity and potential to reverse multidrug resistance produced by *Prochloron didemni*, a cyanobacterial symbiont of the marine ascidian, *Lissoclinum patella*, have been produced through expression in *Escherichia coli* [156,157]. Furthermore, techniques, such as immunofluorescent viability screening and micromanipulation, may improve the efficiency of microbial cultivation [144]. Finally, with the development and increasing affordability of new sequencing technologies, whole genomes/transcriptomes and proteomes from microorganisms may be sequenced and subsequently mined for new bioactive metabolites or to provide relevant information for the development of successful culture methods [155].

The range of both culture-dependent and independent approaches available for the discovery and high-throughput production of new bioactive compounds from symbiotic microorganisms provides excellent prospects that the true potential of these metabolites for drug discovery may soon be unveiled. Together, these methods may allow marine invertebrate symbionts to provide a steady and sustainable supply of bioactive secondary metabolites to fuel the next generation of MNP. 

## 6. Future Prospects

The production of biomass of marine invertebrate-microorganisms assemblages through *in toto* aquaculture is a potential solution to some of the critical challenges that the pharmaceutical industry has been facing in order to find a constant and reliable supply of biomass to fuel the marine drug discovery pipeline. *Ex situ* aquaculture provides a stable environment and allows the application of specific stressors/effects that may trigger metabolic reactions in the microbiota associated with marine invertebrates and promote higher yields of target metabolites. It is important to stress that although aquaculture is not the final solution to solve the “supply issue” in marine drug discovery (from bioprospecting to drug commercialization), it may certainly be a suitable approach for the initial steps of the drug discovery pipeline, namely, while synthetic or semisynthetic alternatives are still being technically and financially optimized. Furthermore, the combination of aquaculture practices with a variety of techniques to cultivate symbiotic microorganisms appears to be a highly promising approach for the discovery of new bioactive compounds from invertebrate-microorganisms assemblages. Culture independent methods may also contribute significantly to marine drug discovery [39], either by helping to improve microbial cultivation techniques or by producing the target metabolite by transgenic organisms able to express the gene responsible for its synthesis.
